# Hypertonic Salt Solution Enhances Inflammatory Responses in Cultured Splenic T-Cells from Dahl Salt-Sensitive Rats but Not Dahl Salt-Resistant Rats

**DOI:** 10.3390/jcdd10100414

**Published:** 2023-10-02

**Authors:** Sungmin Jang, Jee Young Kim, Cheong-Wun Kim, Inkyeom Kim

**Affiliations:** 1 Department of Pharmacology, School of Medicine, Daegu, 41944, Republic of Korea; wkdtjdals6487@naver.com (S.J.); withjyk12@gmail.com (J.Y.K.); kcw7829@nate.com (C.-W.K.); 2 Cardiovascular Research Institute, Daegu 41944, Republic of Korea; 3 BK21 Plus KNU Biomedical Convergence Program, Daegu 41944, Republic of Korea; 4 Department of Biomedical Science, The Graduate School, Kyungpook National University, Daegu 41944, Republic of Korea

**Keywords:** hypertonic salt solution, salt sensitivity, Th17 cells, Treg cells, Dahl salt-sensitive rats, Dahl salt-resistant rats

## Abstract

This study aimed to delineate the effect of sodium chloride on the induction of inflammatory responses and the development of hypertension in Dahl salt-sensitive (SS) and salt-resistant (SR) rats. Splenocytes were isolated from the spleens of SS and SR rats, and cultured on anti-CD3-coated plates for 5 days. The cultured splenic T-cells were challenged with a hypertonic salt solution (0, 20, or 40 mM) in the absence or presence of IL-6 (0, 20, or 60 ng/mL), TGF-β (0, 5, or 15 ng/mL), or IL-23 (0, 10, or 30 ng/mL), and analyzed via ELISA, flow cytometry, and immunofluorescence. The hypertonic salt solution potentiated IL-17A production, as well as the differentiation of Th17 cells via IL-6/TGF-β/IL-23, exclusively in SS rats. However, it did not affect IL-10 production or the differentiation of Treg cells in any of the groups. Furthermore, it potentiated the signal of RORγt in IL-6-treated splenic T-cells from SS rats. To summarize, cultured splenic T-cells exhibited enhanced inflammatory responses on exposure to a hypertonic salt solution in SS rats only, which indicated that sodium chloride and inflammatory cytokines synergistically drove the induction of pathogenic Th17 cells and the development of hypertension in this group only.

## 1. Introduction

Salt affects the tissue microenvironment, organ-specific immune regulation, and autoimmunity [1]. A high salt intake is a major risk factor for the development of hypertension, and it therefore plays an important role in the development of renal and cardiovascular diseases [2]. In the well-established rodent model of salt-sensitive hypertension, Dahl salt-sensitive (SS) and Dahl salt-resistant (SR) rats show different patterns of salt-induced systolic pressure changes. The blood pressure of SS rats rapidly increases, whereas that of SR rats remains normotensive even with a high salt intake [3].

When SR rats consume high amounts of salt, the norepinephrine and sodium channel activities are suppressed, and the diuretic or natriuretic responses to the sodium channels contribute to salt resistance and, thus, improve normotension maintenance [4]. SR rats injected with angiotensin II (Ang II) showed increased nitric oxide (NO) in the thick ascending limbs, resulting in the diffusion of NO into the vasa recta pericyte, but this was not the case in SS rats. Ang II type 2 receptors in SS rats are defective, and reactive oxygen species (ROS), which regulate the differentiation of T helper (Th) 17 cells, have become more dominant over NO in SR rats [5,6]. IL-17A, which is mainly secreted by Th17 cells, affects renal sodium retention, regulates pressure natriuresis, and enhance vascular superoxide production but also attenuates the phosphorylation of endothelial nitric oxide synthase. Therefore, inflammatory cytokines reduce the diameter of blood vessels, leading to the development of hypertension [7].

In our previous studies, these two groups of rats also showed differences in blood pressure changes with a high salt intake as well as with a high fructose intake [8], high fat intake [9], and Ang II infusion [10]. A high fructose intake or recombinant IL-23 injection increased the population of Th17 cells and the serum levels of IL-17A in SS rats; however, a high population of Treg cells and increased serum IL-10 levels in the SR rats protected them from hypertension [8]. A high fat intake did not affect the expression of adipocyte marker genes in the thoracic perivascular adipose tissue or the renin–angiotensin–aldosterone system in the kidneys of the SR rats compared to the SS group [9]. The Ang II (50 ng/kg/min) infusion induced mRNA expression related to Th17 cells and increased the mRNA expression of *Il-6* and *Il-1β*, which activated the inflammatory response in SS rats but not in SR rats. Moreover, SR rats have a high basal mRNA expression of transforming growth factor β (*Tgf-β*), which upregulates the expression of forkhead box P3 (*Foxp3*) [10].

In chronic inflammatory diseases, cytokines such as IL-6, IL-1β, and tumor necrosis factor-*α* (TNF-*α*) have the potential to exacerbate excessive cardiovascular risk [11]. IL-6 recruits Janus kinase 1 (JAK1), which phosphorylates signal transducers and activators of transcription 3 (STAT3). Phosphorylated STAT3 (pSTAT3) induces the expression of Th17-related genes and the master transcription factor RORγt [12,13]. In addition to IL-6, IL-23 plays a pivotal role in maintaining the Th17 phenotype [14]. The function of TGF-β in the induction of immune responses is controversial, with TGF-β being able to switch between pro-inflammatory and anti-inflammatory responses in the presence or absence of the IL-6 cytokine. In the absence of IL-6, TGF-β is indispensable in Treg cell differentiation and FoxP3 activation [15].

This study aimed to explore whether different concentrations of IL-6 and TGF-β could affect the differentiation of Th17 or Treg cells in the presence of a hypertonic salt solution in cultured splenic T-cells from SS and SR rats. The addition of IL-23, which preferentially induces the differentiation of Th17 cells, was also investigated for the differentiation of Th17 or Treg cells in cultured splenic T-cells from SS and SR rats. Furthermore, we tested the hypothesis that a hypertonic salt solution enhances inflammatory responses in cultured splenic T-cells from Dahl salt-sensitive rats, but not in Dahl salt-resistant rats.

## 2. Materials and Methods

### 2.1. Animals

The research study adhered to the guidelines outlined in the National Institutes of Health Guide for the Care and Use of Laboratory Animals and received approval from the Institutional Review Board of Kyungpook National University (Approval No. 2021-0192). Great care was taken to minimize both the number of animals involved and any potential suffering. Male SS (DIS/EisSlc, Dahl-Iwai S) and SR (DIR/EisSlc, Dahl-Iwai R) rats, aged seven weeks, were obtained from Japan SLC Inc. (Hamamatsu, Shizuoka, Japan, *n* = 6) and underwent a two-week acclimatization period. The rats were anesthetized with sodium pentobarbital (50 mg/kg intraperitoneally, provided by Hanlim Pharm, Co., Ltd., Yongin, Republic of Korea) for humane sacrifice. Following anesthesia, tissues were obtained, frozen in liquid nitrogen, and stored at −80 °C for further study.

### 2.2. Cell Culture

**Figure 1:**
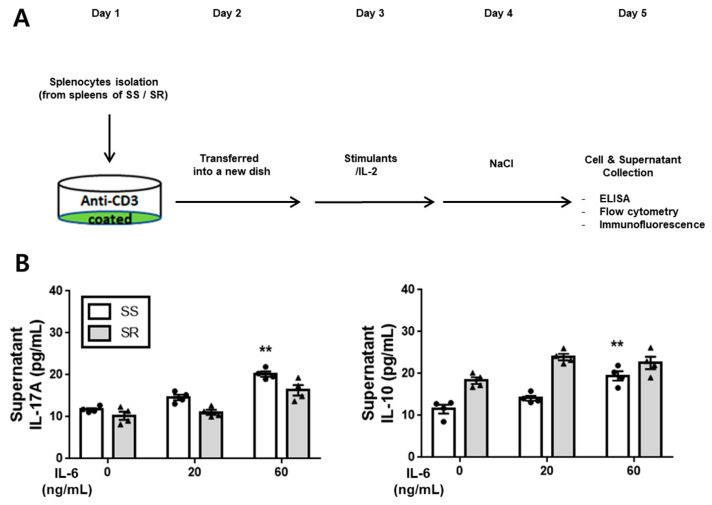

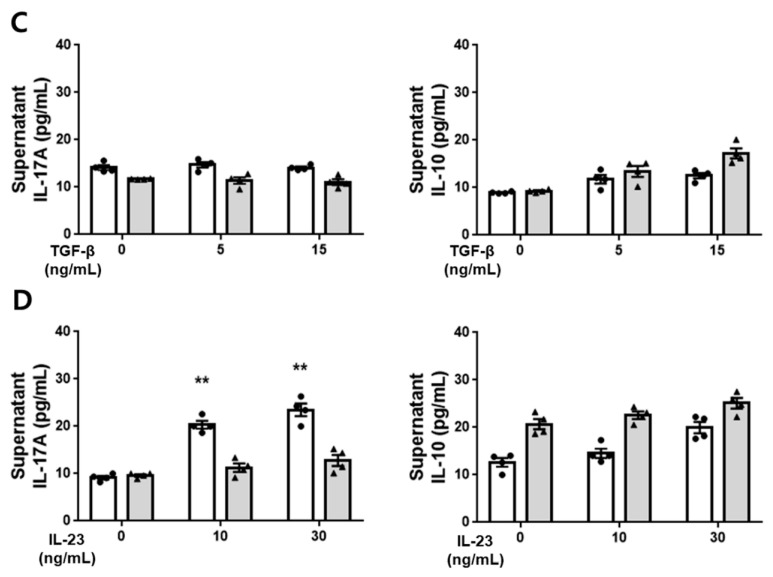
(**A**) The experimental design overview. (**B**–**D**) The quantification of the IL-17A and IL-10 levels in the supernatants of cultured splenic T-cells, assessed via ELISA. (**B**) Elevated levels of IL-6 resulted in an increased production of both IL-17A and IL-10 in cultured splenic T-cells from SS rats, while no such effect was observed in SR rats. (**C**) The presence of TGF-β had no significant impact on the production of IL-17A and IL-10 in cultured splenic T-cells from either SS or SR rats. (**D**) IL-23 induced a concentration-dependent rise in IL-17A production in the cultured splenic T-cells from SS rats, but had no such effect on those from SR rats. The mean values ± SEM are presented for four independent experiments. Two-way ANOVA, followed by Tukey’s post hoc multiple comparison test, was utilized for statistical analysis. ** *p* < 0.01 compared to the respective control samples.

The cell culture protocol, as depicted in Figure 1A, involved the isolation of splenocytes from the spleens both of Dahl salt-sensitive (SS) and of salt-resistant (SR) rats. These splenocytes, at a density of 3 × 10^6^ cells per well in 6-well plates, were cultivated in RPMI-1640 medium. The medium was supplemented with 10% fetal bovine serum (FBS), 1% sodium pyruvate, 50 µg/mL of penicillin and streptomycin, 50 µM β-mercaptoethanol, and 25 mM HEPES, all sourced from Gibco (Carlsbad, CA, USA). The 6-well plates had been pre-coated with 4 µg/mL of anti-CD3 antibody (clone G4.18, #554830, BD Bioscience, Franklin Lakes, NJ, USA) [16]. On the following day, the cells (1 × 10^6^ cells per well in 6-well plates) were transferred to new plates. To initiate cell differentiation, a differentiation cocktail comprising 2 µg/mL of anti-CD28 antibody (#559982, BD Bioscience, Franklin Lakes, NJ, USA), 5 ng/mL of recombinant rat transforming growth factor β (TGF-β, G11622, LSBio, Seattle, WA, USA), 20 ng/mL of recombinant rat IL-6, and 2 ng/mL of recombinant rat IL-2 (obtained from R&D Systems, Minneapolis, MN, USA) was used for treatment. To assess the impact of hypertonic salt solutions, cultured splenic T-cells were exposed to the additional NaCl concentrations of 0, 20, or 40 mM (CAS 7647-14.5, Junsei, Japan) [1]. The collection of both the cultured cells and the supernatants was performed on the fifth day for subsequent enzyme-linked immuno-sorbent assay (ELISA), flow cytometry, or immunocytochemistry-based analyses.

### 2.3. Measurement of Cytokines in Supernatants of Cultured Splenic T-Cells

The levels of IL-17A (BMS635, Thermo Fisher Scientific, Waltham, MA, USA) and IL-10 (R1000, R&D Systems, Minneapolis, MN, USA) in the supernatants of the cultured splenic T-cells were quantified using ELISA kits. The optical density was measured at a wavelength of 450 nm, and the concentrations of IL-17A and IL-10 were determined through reference to standard curves.

### 2.4. Flow Cytometry Analysis of Cultured Splenic T-Cells

For the performance of the phenotypic analyses, the cultured splenic T-cells were incubated with specific antibodies targeting T-cell surface markers. This included fluorescein (FITC)-conjugated anti-CD3 (1:200, clone G4.18, #554832, BD Bioscience, Franklin Lakes, NJ, USA) and phycoerythrin (PE)-conjugated anti-CD4 (1:200, clone OX-35, #554838, BD Bioscience). For the identification of Treg and Th17 cells, the cells were stained with peridinin-chlorophyll-protein (PerCP)–Cyanine5.5 conjugated anti-FoxP3 (1:100, clone FJK-16s, #45-5773-82, Thermo Fisher Scientific) for Treg cells or allophycocyanine (APC)-conjugated anti-retinoic-acid-receptor-related orphan nuclear receptor gamma (RORγt) (1:1000, clone 4G419, NBP2-24449, Novus Biologicals, Centennial, CO, USA) for Th17 cells, employing the FoxP3/Transcription Factor Staining Buffer Set (00-5523-00, eBioscience) [17]. The data were acquired using a 4-color flow cytometer (FACS Calibur; BD Bioscience, Franklin Lakes, NJ, USA) and were subsequently analyzed using FlowJo v10 software (BD Biosciences).

### 2.5. Immunofluorescence

The cultured splenic T-cells were initially fixed using a 4% paraformaldehyde solution and were subsequently permeabilized according to the manufacturer’s instructions provided in the FoxP3/Transcription Factor Staining Buffer set (00-5523-00, eBioscience, San Diego, CA, USA) [18]. Following permeabilization, the cells were subjected to blocking using 5% bovine serum albumin (BSA) and then incubated with a 1:1000 dilution of rabbit anti-human RORγt (orb385620, Biorbyt, Cambridge, UK). Subsequently, they were treated with goat anti-rabbit IgG (H+L) cross-adsorbed Alexa Fluor™ 594 secondary antibody (1:2000, a11012, ThermoFisher Scientific) diluted in permeabilization buffer containing 5% BSA. DAPI (50 ng/mL, 62248; Thermo Fisher Scientific) was used for co-staining. The immunocytochemistry images were captured using a fluorescence microscope (40×, DMI3000 B, Leica, Germany).

### 2.6. Statistics

The data are expressed as mean ± standard error of mean (SEM). Statistical analyses were performed with the two-way or multiple ANOVA method followed by Tukey’s post hoc multiple comparison test using GraphPad Prism 7 (GraphPad Software, San Diego, CA, USA), with a *p*-value of <0.05 being considered significant.

## 3. Results

### 3.1. IL-6 and IL-23 Increased the Production of IL-17A in Isotonic Salt Solution in the Supernatants of Cultured Splenic T-Cells from SS Rats, but Not SR Rats

Cultured splenic T-cells from SS and SR rats were subjected to varying concentrations of IL-6, TGF-β, and IL-23 in the presence of an isotonic salt solution. The subsequent measurements of IL-17A and IL-10 levels in cell supernatants were performed using ELISA. The results showed that IL-6 (at a concentration of 60 ng/mL) increased the production of both IL-17A and IL-10 exclusively in SS-rat-derived splenic T-cells (Figure 1B). Conversely, TGF-β had no discernible effect on the production of IL-17A or IL-10 in splenic T-cells from either SS or SR rats (Figure 1C). Notably, IL-23 boosted the production of IL-17A only in SS-rat-derived splenic T-cells, while it had no impact on IL-10 production in either the SS or SR group (Figure 1D).

### 3.2. IL-6 and IL-23 Increased the Production of IL-17A in Isotonic Salt Solution in the Supernatants of Cultured Splenic T-Cells from SS Rats, but Not SR Rats

**Figure 2:**
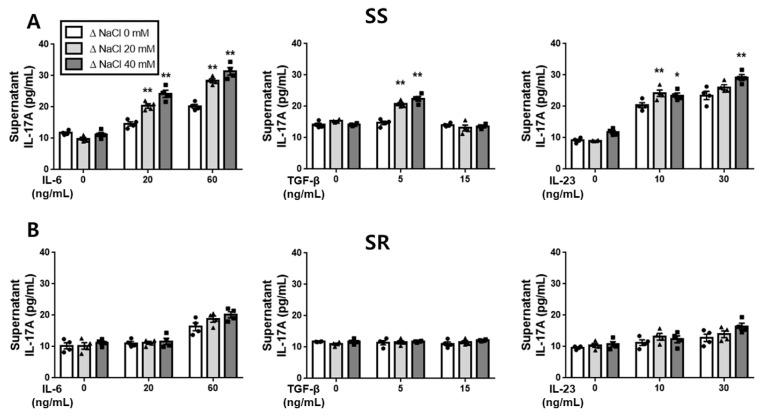
The enhanced IL-17A production in cultured splenic T-cells exposed to a hypertonic salt solution. (**A**) The influence of the hypertonic salt solution on IL-17A production triggered by IL-6, TGF-β (up to 5 ng/mL), or IL-23 in the supernatants of cultured splenic T-cells from SS rats. (**B**) No significant impact of the hypertonic salt solution on IL-17A production was observed in the cultured splenic T-cells from SR rats. The IL-17A levels in the supernatants of the cultured splenic T-cells from both SS and SR rats were quantified using ELISA. The data represent mean ± SEM from four independent experiments. Statistical analysis was performed using multiple ANOVA, followed by Tukey’s post hoc multiple comparison test. * *p* < 0.05 and ** *p* < 0.01 compared to the corresponding isotonic control conditions.

**Figure 3:**
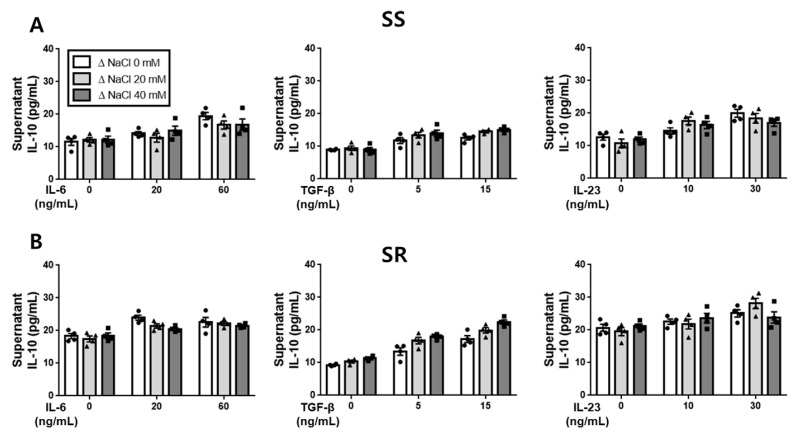
The impact of a hypertonic salt solution on IL-10 production in the cultured splenic T-cells. (**A**) The evaluation of the influence of a hypertonic salt solution on the IL-10 production stimulated by IL-6, TGF-β, or IL-23 in the supernatants of the cultured splenic T-cells from SS rats. (**B**) A similar assessment in the supernatants of the cultured splenic T-cells from SR rats revealed no significant modulation of IL-10 production by the hypertonic salt solution. The levels of IL-10 in the cell supernatants from both SS and SR rats were quantified using ELISA. The presented values represent the mean ± SEM from four independent experiments. The statistical analysis involved multiple ANOVA, followed by Tukey’s post hoc multiple comparison test.

We examined the influence of a hypertonic salt solution on the production of IL-17A and IL-10 in the presence of IL-6, TGF-β (up to 5 ng/mL), or IL-23 in the cell supernatants of cultured splenic T-cells derived from SS and SR rats. The hypertonic salt solution exhibited a potentiating effect on IL-17A production induced by IL-6, TGF-β, or IL-23 exclusively in splenic T-cells from the SS rats, while it had no such effect in the SR rats (Figure 2). Conversely, the hypertonic salt solution did not exert any impact on the IL-10 production induced by any of the cytokines in the cell supernatants of the cultured splenic T-cells from either the SS or SR rat group (Figure 3).

### 3.3. Effect of Hypertonic Salt Solution on the Differentiation of Th17 Cells in Cultured Splenic T-Cells from SS Rats and SR Rats

**Figure 4:**
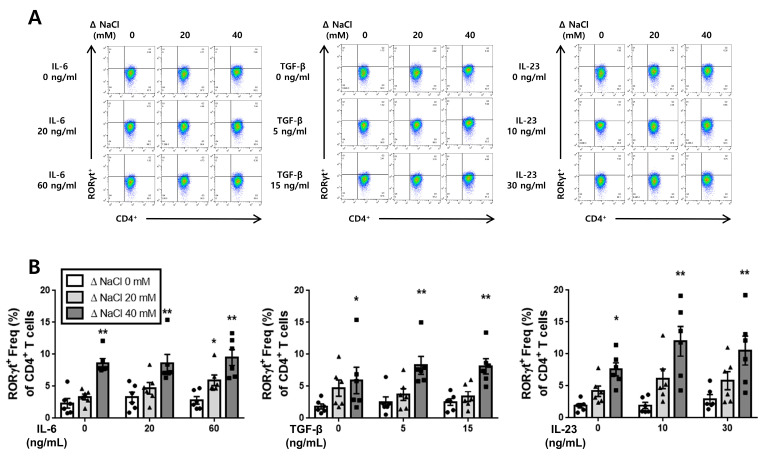
The influence of a hypertonic salt solution on Th17 cell differentiation in cultured splenic T-cells from SS rats. (**A**,**B**) Representative flow cytometry plots and accompanying bar graphs with scatter plots illustrating the frequency of Th17 cells (CD4^+^RORγt^+^) in the cultured splenic T-cells from SS rats. The hypertonic salt solution was found to enhance the differentiation of Th17 cells in these cultured splenic T-cells. The data presented represent the mean ± SEM derived from six independent experiments. The statistical analysis involved multiple ANOVA, followed by Tukey’s post hoc multiple comparison test. * *p* < 0.05 and ** *p* < 0.01 compared to the corresponding isotonic control conditions.

**Figure 5:**
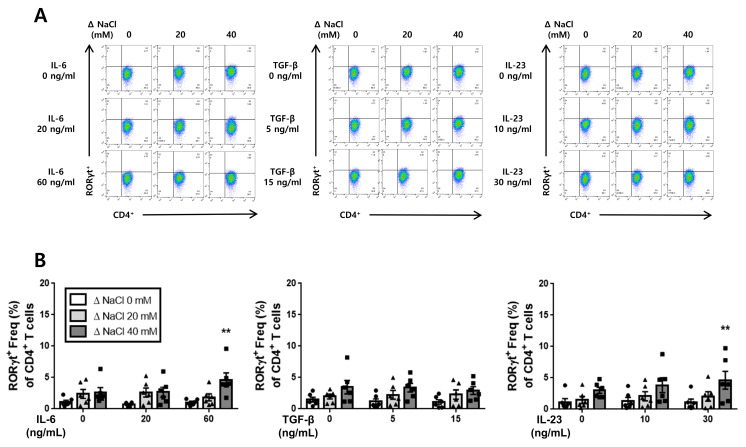
The enhanced Th17 cell differentiation in the cultured splenic T-cells from SR rats with a hypertonic salt solution. (**A**,**B**) Illustration of representative flow cytometry plots and associated bar graphs depicting the frequency of Th17 cells (CD4^+^RORγt^+^) in cultured splenic T-cells from SR rats. The presence of the hypertonic salt solution resulted in an increased differentiation of Th17 cells when combined with high concentrations of IL-6 (60 ng/mL) or IL-23 (30 ng/mL) in the cultured splenic T-cells from SR rats. The data presented represent the mean ± SEM from six independent experiments. The statistical analysis involved multiple ANOVA, followed by Tukey’s post hoc multiple comparison test. ** *p* < 0.01 compared to the corresponding isotonic control conditions.

Following the obtention of the ELISA results, we conducted flow cytometry analysis to validate the differentiation of cultured splenic T-cells into Th17 cells in both the SS and the SR rats. The flow cytometry results revealed that the hypertonic salt solution increased the population of Th17 cells (CD4^+^RORγt^+^) in the presence of IL-6, TGF-β, or IL-23 specifically in SS rats (Figure 4). Conversely, in the cultured splenic T-cells from SR rats, the hypertonic salt solution was found to enhance the Th17 cell population only at higher concentrations of IL-6 (60 ng/mL) or IL-23 (30 ng/mL) (Figure 5).

### 3.4. Hypertonic Salt Solution Did Not Affect the Differentiation of Treg Cells in Cultured Splenic T-Cells from SS Rats and SR Rats

**Figure 6:**
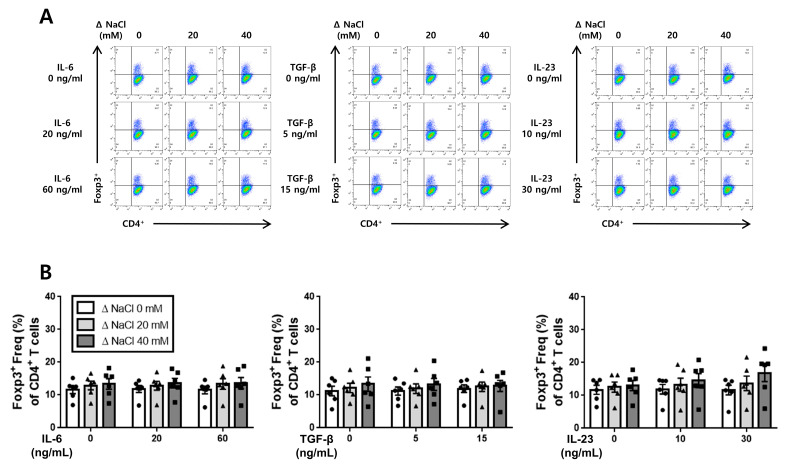
The influence of the hypertonic salt solution on Treg cell differentiation in the cultured splenic T-cells from SS rats. (**A**,**B**) Depiction of the representative flow cytometry plots and accompanying bar graphs featuring the frequency of Treg cells (CD4^+^Foxp3^+^) in the cultured splenic T-cells from SS rats. Regardless of the increasing concentration of IL-6, TGF-β, or IL-23, the hypertonic salt solution did not demonstrate any significant influence on the differentiation of Treg cells in the cultured splenic T-cells from SS rats. The data presented represent the mean ± SEM derived from six independent experiments. The statistical analysis involved multiple ANOVA, followed by Tukey’s post hoc multiple comparison test.

**Figure 7:**
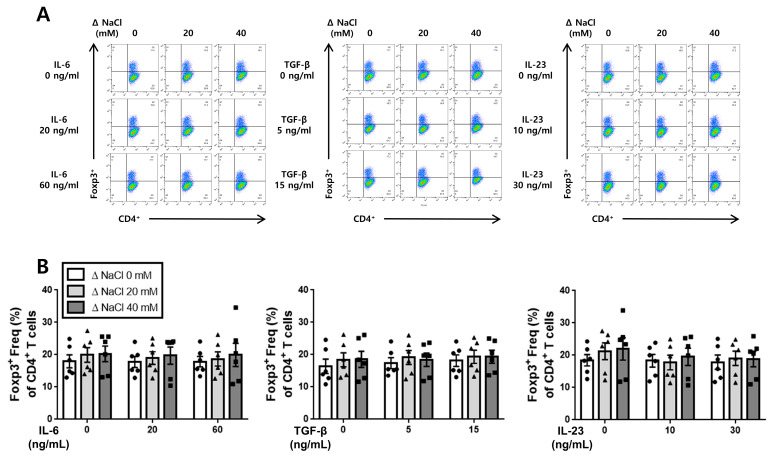
The impact of hypertonic salt solution on Treg cell differentiation in the cultured splenic T-cells from the SR rats. (**A**,**B**) Presentation of the representative flow cytometry plots and accompanying bar graphs, featuring the frequency of Treg cells (CD4^+^Foxp3^+^) in the cultured splenic T-cells from the SR rats. Irrespective of the increasing concentration of IL-6, TGF-β, or IL-23, the presence of the hypertonic salt solution did not exert any significant effect on the differentiation of Treg cells in the cultured splenic T-cells from the SR rats. The data presented represent the mean ± SEM derived from six independent experiments. The statistical analysis involved multiple ANOVA, followed by Tukey’s post hoc multiple comparison test.

We investigated whether the hypertonic salt solution had any impact on the differentiation of Treg cells in cultured splenic T-cells from both the SS and the SR rats, comparing it to the differentiation of Th17 cells. Our findings indicate that the hypertonic salt solution did not influence the differentiation of Treg cells when induced by IL-6, TGF-β, or IL-23 in the cultured splenic T-cells from both the SS and the SR rats (Figure 6 and Figure 7).

### 3.5. Immunofluorescence Analysis Indicated That the Hypertonic Salt Solution Resulted in an Increase in Th17 Cells in the Presence of IL-6 in Cultured Splenic T-Cells from Only the SS Rats and Not the SR Rats

**Figure 8:**
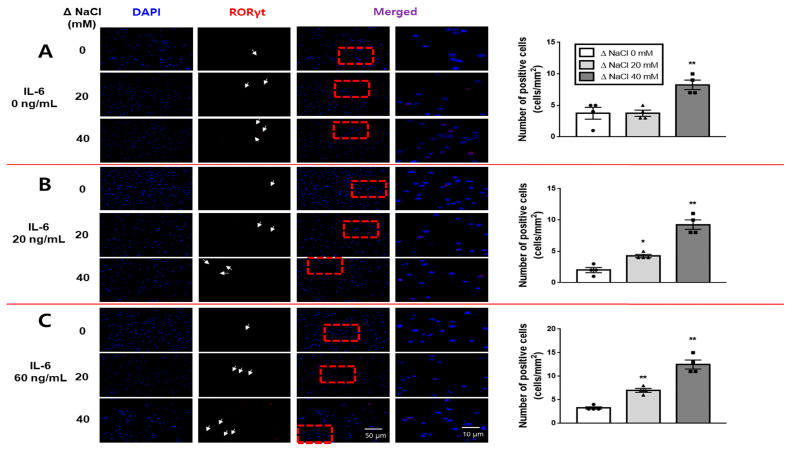
Immunofluorescence analysis of the impact of the hypertonic salt solution on Th17 cell differentiation in cultured splenic T-cells from SS rats. (**A**–**C**) Presentation of representative immunofluorescence images accompanied by bar graphs quantifying the number of RORγt-positive cells in cultured splenic T-cells from SS rats. The cultured splenic T-cells were subjected to either isotonic or hypertonic salt solutions (0, 20, or 40 mM NaCl) in the presence of escalating concentrations of IL-6. The scale bar for 40× magnification was set at 50 μm. The quantification of RORγt-positive cells (cells/mm^2^, depicted in red), alongside DAPI (blue), was performed manually. The hypertonic salt solution exhibited an enhancing effect on the RORγt signal in cultured splenic T-cells from SS rats, especially in conjunction with increasing concentrations of IL-6. The data presented represent the mean ± SEM derived from four independent experiments. The statistical analysis involved ANOVA, followed by Tukey’s post hoc multiple comparison test. * *p* < 0.05 and ** *p* < 0.01 compared to the corresponding isotonic control conditions.

**Figure 9:**
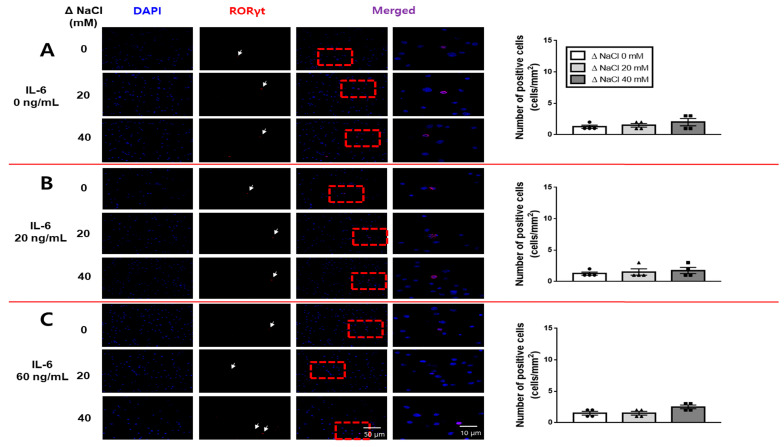
Immunofluorescence analysis of the impact of a hypertonic salt solution on Th17 cell differentiation in cultured splenic T-cells from SR rats. (**A**–**C**) Presentation of representative immunofluorescence images alongside bar graphs quantifying the number of RORγt-positive cells in the cultured splenic T-cells from SR rats. The cultured splenic T-cells were exposed to either an isotonic or hypertonic salt solution (0, 20, or 40 mM NaCl) in the presence of increasing concentrations of IL-6. The scale bar for 40× magnification was set at 50 μm. The manual quantification of RORγt-positive cells (cells/mm^2^, represented in red), in conjunction with DAPI (blue), was conducted for each experimental condition. Neither the hypertonic salt solution nor the escalating concentrations of IL-6 demonstrated a significant effect on the RORγt signal in the cultured splenic T-cells from SR rats. The data presented represent the mean ± SEM derived from four independent experiments. The statistical analysis involved ANOVA, followed by Tukey’s post hoc multiple comparison test.

We employed immunofluorescence analysis to investigate whether the hypertonic salt solution led to an increase in Th17 or Treg cells in the presence of IL-6 within cultured splenic T-cells from both the SS and the SR rats. The results showed that the hypertonic salt solution specifically increased the population of RORγt-positive (Th17) cells at elevated concentrations of IL-6 in cultured splenic T-cells from SS rats (Figure 8), while it did not have the same effect in SR rats (Figure 9).

## 4. Discussion

This study proves that a hypertonic salt solution enhances inflammatory responses in cultured splenic T-cells from SS rats, but not those from SR rats. The concentrations of hypertonic salt solutions are 20 and 40 mM, the latter of which increases Th17 cells in rodent and human cells [1,14]. IL-6 and IL-23 increased the IL-17A production in the supernatants of the cultured splenic T-cells from SS rats. Notably, the hypertonic salt solution potentiated the production of IL-17A by IL-6, TGF-β, or IL-23 in the supernatants of cultured splenic T-cells from SS rats but not those from SR rats. The hypertonic salt solution also increased the differentiation of Th17 cells in the presence of IL-6, TGF-β, or IL-23 in cultured splenic T-cells from SS rats, but not those from SR rats. However, the hypertonic salt solution did not affect IL-10 production and Treg cell differentiation in the cultured splenic T-cells from either SS or SR rats. Likewise, the cultured splenic T-cells from SS and SR rats showed differential immune responses to the hypertonic salt solution or cytokines. Different molecular mechanisms cause differences in immune responses between SS and SR rats in response to increasing salt concentrations, leading to the development of inflammatory diseases, such as hypertension [19,20].

It has been reported that the differentiation of Th17 cells can be a driven by a cytokine cocktail (IL-1β, IL-6, IL-21, IL-23, and TGF-β1), and a hypertonic salt solution promotes the induction of Th17 cells [1]. In our study, increasing concentrations of IL-6 increased the production of IL-17A in the isotonic solution from SS rats, but not from SR rats. IL-6 signaling induces the development of Th17 cells by programming Th17-related genes via STAT3 and plays a significant role in inflammatory pathogenesis [21,22,23]. IL-6, in the hypertonic salt solution, potentiates the differentiation of Th17 cells in comparison to the isotonic salt solution [14]. The hypertonic salt solution increased the production of IL-17A in the supernatants of the cultured splenic T-cells, the differentiation of Th17 cells as well as the RORγt signal in the cultured splenic T-cells from SS rats, compared to the isotonic salt solution. The hypertonic salt solution with the highest concentration of IL-6 induced the differentiation of Th17 cells in the cultured T-cells from SR rats. Therefore, IL-6 has been proven to be necessary for the differentiation of Th17 cells, which was potentiated via a hypertonic salt solution.

We also demonstrated a role for TGF-β in the production of IL-17A, as well as the differentiation of Th17 cells in cultured splenic T-cells from SS and SR rats. TGF-β did not affect the production of either IL-17A or IL-10 in the isotonic solution from SS and SR rats, whereas TGF-β (5 ng/mL), along with the hypertonic salt solution, potentiated the production of IL-17A from SS rats. Although TGF-β induces the expression of FoxP3 and has been reported to suppress inflammatory responses [24,25], it can induce the differentiation of Th17 cells along with IL-6 in the presence of a hypertonic salt solution [16]. Therefore, TGF-β can induce either anti-inflammatory or pro-inflammatory T-cells in the presence or absence of pro-inflammatory cytokines, such as IL-6 and IL-23, or a hypertonic salt solution [26,27].

Previous research has reported that IL-23 plays a pivotal role in inducing the differentiation of T helper cells into Th17 cells [28]. In accordance with these findings, a prior study demonstrated that IL-23 not only increases the differentiation of Th17 cells but also potentiates this differentiation in the presence of a hypertonic salt solution [14]. Given these insights, our study sought to investigate whether IL-23 exerts any discernible influence on the differentiation of Th17 cells in cultured splenic T-cells derived from both SS and SR rats.

In our study, while IL-23 increased the production of IL-17A in the supernatants of cultured splenic T-cells from SS rats in an isotonic salt solution, it increased the differentiation of Th17 cells in cultured splenic T-cells from SS rats in the presence of a hypertonic salt solution, thereby inducing the production of IL-17A in the cultured supernatants from SS rats, but not those from SR rats. IL-23, at its highest concentration in the hypertonic salt solution, induced the differentiation of Th17 cells in the cultured T-cells from SR rats. IL-23, which maintains the differentiation of Th17 cells, is associated with high serum/glucocorticoid-regulated kinase 1 (SGK1) expression in a high-salt microenvironment. The high SGK1 expression activates the subsequent induction of the RORγt signal [14], but suppresses FoxP3 expression by phosphorylating FOXO1 [29]. However, in our results, the production of IL-10 in the supernatant and the differentiation of Treg cells in cultured splenic T-cells did not change in the hypertonic salt solution with various concentrations of cytokines from SS and SR rats. IL-6 promotes the development of Th17 cells but inhibits Treg cells, whereas IL-2 promotes the development of Treg cells but inhibits Th17 cells [23]. Therefore, our results suggest that the use of IL-2 may have an impact on the production of IL-10 and the differentiation of Treg cells in a high-salt solution.

In our in vitro experiments using splenocytes from SS rats, we meticulously confirmed the robust generation of IL-17A and the differentiation of Th17 cells in response to a hypertonic salt solution and various cytokines. Interestingly, the results for the SR rats exhibited marked differences, with a notably weaker response compared to the SS rats. Notably, there were no significant differences observed in the IL-17A levels based on varying hypertonic salt solution concentrations and cytokine dosages. While there was an evident increase in pro-inflammatory Th17 cells, the response appeared comparatively less pronounced compared to the robust response observed in the SS rats. These intriguing findings were subsequently validated through in vivo experiments [30]. Through these comprehensive experiments, we have successfully identified IL-17A and Th17 cell levels as promising potential biomarkers for salt-induced hypertension. These discoveries hold great promise in advancing early diagnosis and, thus, contribute significantly to the prevention and treatment of hypertension.

## 5. Conclusions

The hypertonic salt solution enhances inflammatory responses in cultured splenic T-cells from SS, but not SR rats. SS and SR rats have genetic differences that determine the balance between Th17 and Treg cells based on the hypertonic salt solution and the presence of cytokines. Therefore, the different molecular levels in SS and SR rats exposed to a hypertonic salt solution along with cytokines may be related to the development of hypertension and inflammatory diseases.

## Data Availability

Not applicable.
